# Laypersons' understanding of relative risk reductions: Randomised cross-sectional study

**DOI:** 10.1186/1472-6947-8-31

**Published:** 2008-07-17

**Authors:** Lene Sorensen, Dorte Gyrd-Hansen, Ivar S Kristiansen, Jørgen Nexøe, Jesper B Nielsen

**Affiliations:** 1 Amgros I/S, Copenhagen, Denmark; 2 Institute of Public Health, University of Southern Denmark, Odense, Denmark; 3 Institute of Health Management and Health Economics, University of Oslo, Norway

## Abstract

**Background:**

Despite increasing recognition of the importance of involving patients in decisions on preventive healthcare interventions, little is known about how well patients understand and utilise information provided on the relative benefits from these interventions. The aim of this study was to explore whether lay people can discriminate between preventive interventions when effectiveness is presented in terms of relative risk reduction (RRR), and whether such discrimination is influenced by presentation of baseline risk.

**Methods:**

The study was a randomised cross-sectional interview survey of a representative sample (n = 1,519) of lay people with mean age 59 (range 40–98) years in Denmark. In addition to demographic information, respondents were asked to consider a hypothetical drug treatment to prevent heart attack. Its effectiveness was randomly presented as RRR of 10, 20, 30, 40, 50 or 60 percent, and half of the respondents were presented with quantitative information on the baseline risk of heart attack. The respondents had also been asked whether they were diagnosed with hypercholesterolemia or had experienced a heart attack.

**Results:**

In total, 873 (58%) of the respondents consented to the hypothetical treatment. While 49% accepted the treatment when RRR = 10%, the acceptance rate was 58–60% for RRR>10. There was no significant difference in acceptance rates across respondents irrespective of whether they had been presented with quantitative information on baseline risk or not.

**Conclusion:**

In this study, lay people's decisions about therapy were only slightly influenced by the magnitude of the effect when it was presented in terms of RRR. The results may indicate that lay people have difficulties in discriminating between levels of effectiveness when they are presented in terms of RRR.

## Background

Patient autonomy is a core element of medical ethics. Patient autonomy implies that patients and doctors share responsibility for medical decision to the extent patients wish to be included. For shared decision making to be meaningful, however, patients need to have an understanding of the effectiveness of medical interventions. This usually requires the use of the risk concept. Communicating risk information is therefore a fundamental and increasingly prominent part of medical practice. Effective risk communication can enhance knowledge, involvement in decisions about testing or treatment, autonomy and empowerment of patients [1]. However poor communication may possibly lead to anxiety or lack of confidence in health care professionals [2]. It is vital that we identify the available evidence about how risk communication should best be done.

The effectiveness of an intervention for a chronic disease may be presented in terms of Relative Risk Reduction (RRR), Absolute Risk Reduction (ARR), Numbers Needed to Treat (NNT), or Odds Ratio (OR). These measures may subsequently be translated into increased (disease-free) life expectancy because the intervention postpones adverse events. The choice of effect measure has been discussed extensively in the scientific literature (e.g. by Gigerenzer et al [3] or Elmore et al [4]), but we still need more knowledge about how intervention effectiveness can be communicated to patients, doctors and health administrators.

Decisions should, in line with expected utility theory [5], be based on the absolute risk reductions (or its reciprocal NNT) even though this principle in practice is frequently violated. However, there is evidence that lay-persons and professionals may have difficulties in understanding NNT [6-8]. Great effectiveness of a treatment corresponds with low value of NNT, and this may mislead patients if they associate great effectiveness with a large number. In contrast, for RRR a greater value means greater effectiveness. RRR, however, may be misleading because it usually is greater than ARR numerically, and may consequently "exaggerate" the treatment effect. Still, RRR is frequently presented in the medical literature, possibly because it is more stable across patient groups than ARR [9]. It is therefore conceivable that doctors on some occasions use RRR in their communication with patients. Even though decisions should not be based on RRR alone, we need to know to which extent patients can utilise information about RRR. We have searched the literature without finding any direct evidence of the extent to which lay people understand RRR.

The size of baseline risk has been shown to influence acceptance of a hypothetical treatment [8]. Usually, baseline risk is presented to supplement RRR to allow for calculation of ARR. In the current study, we tested whether presence or absence of baseline risk information, provided in addition to RRR information, affects acceptance of a hypothetical treatment. In theory, absence of baseline risk leaves the respondent without sufficient information to properly decide on whether or not to accept a treatment, because ARR cannot be derived. Real life decisions are, nevertheless often made without sufficient information [10,11].

We hypothesised that respondents are sensitive to the magnitude of RRR in decisions about a preventive therapy, i.e. respondents are able to differentiate between low and high levels of RRR. We also hypothesised that numeric presentation of baseline risk will reduce respondents' general acceptance of the hypothetical treatment for all levels of RRR, because the presentation of this added piece of information allows the respondent to assess the absolute effect of the intervention. The aim of this study was to test these hypotheses in a sample of laypersons.

## Methods

A random sample of non-institutionalised Danes aged 40+ years was interviewed face-to-face through computer-assisted personal interviews by Gallup Inc, Denmark. Questions were concurrently presented to the respondents orally and in writing on cards.

A sample of 3,548 individuals aged 40+ years was randomly drawn from a national database at Statistics Denmark. The sampling ensured geographic representativity. A total of 415 individuals could not be contacted because they were either non fluent in Danish (n = 62), suffered from sickness, senile dementia or reduced hearing (n = 154) or because the address identified was non-inhabited, non-existing or used for industrial purposes (n = 199)). The net sample consisted of 3,133 individuals. After three attempts, 731 potential respondents could still not be contacted, and 883 refused participation. All together 1,519 (49%) respondents completed an interview.

Information on age, gender, marital status, education and household income was collected. The two latter variables were included, because it is conceivable that understanding of risk information is higher in population groups with higher income or education. Respondents were also asked whether they were diagnosed with hypercholesterolaemia or had experienced a heart attack.

Additionally, the respondents were asked to consider a hypothetical intervention (see below). Similar questions have been used in previous studies conducted by Odense Risk Group, wherefore no pilot testing was performed. In order to test whether baseline risk had an effect on the acceptance of this intervention presented in terms of RRR, respondents were allocated to alternative versions of a case scenario in which baseline numeric risk information was either present or not. The following wording was used:

• Version 1:"Imagine that your GP tells you that you have a slightly increased risk of suffering a heart attack. On average, 10 out of 1000 patients like you will die of a heart attack within 3 years."

• Version 2:"Imagine that your GP tells you that you have a slightly increased risk of suffering a heart attack."

Subsequently, the following information was given to all respondents:

Your GP presents you with a medication, which should be taken once a day. The medication has mild and harmless side effects. The treatment requires that you visit your GP twice a year for a check-up. The annual cost of your medication is approximately 500 DKK (~£45), which you will have to pay yourself.

Your GP tells you that the use of the medication for 3 years will reduce your risk of heart attack by X%."

The impact on choice of the magnitude of RRR was tested by randomly allocating respondents to varying X = 10, 20, 30, 40, 50, 60. We chose these levels because most medical interventions attain effectiveness within this range. RRR was presented as percentages because this is the way they are usually presented in the medical literature even though relative frequencies may be easier to understand [3]. By random, half of the respondents in each RRR group were presented with the baseline risk of heart attack, while the others were not. The randomisation was done by a computer at the start of the interview.

Subsequently, the respondents were asked whether they would choose to take the medication, and also asked to answer a question regarding their perceived difficulty of understanding the RRR information. The following preset answer categories were presented:

"Was it difficult to understand the size of the treatment effect?"

• Not difficult to understand

• A little difficult to understand

• Very difficult to understand

• Impossible to understand

In the subsequent analyses, the responses were recoded into a dichotomous variable, where one category represented those who had no difficulties understanding the case, and the other category represented the remaining respondents.

Variation in consent with increasing RRR was tested with bivariate trend analysis. Additionally, logistic regression was performed to explore determinants of consent including presentation of baseline risk.

With 125 respondents in each RRR-group, we had a power of 99% to detect a trend describing an increase from 40% to 75% acceptance of therapy with increasing effectiveness. A similar effect has been observed in a study of prolongation of life as measure of benefit [12].

## Results

The mean age of the 1,519 respondents was 59.6 years, and 53.9% were women. Age, gender and geographic location of the respondents were similar to that of the background population in Denmark, according to Gallup Inc, who performed the selection of the respondents. Previous heart attack was reported by 6% of the respondents, and 19% reported hypercholesterolaemia, and both values were similar to measures found in the background population. After randomising respondents into the different RRR information groups, no differences were found between the groups with regard to age, household income, gender and prevalence of hypercholesterolaemia or previous heart attack (Table 1).

**Table 1:** Characteristics of respondents in each group

	**RRR = 10**	**RRR = 20**	**RRR = 30**	**RRR = 40**	**RRR = 50**	**RRR = 60**	**All**
Age (years): mean median (minimum-maximum)	59.5 58.0 (40–93)	59.0 58.0 (40–90)	59.6 58.0 (40–91)	60.3 58.0 (40–98)	59.7 57.0 (40–90)	59.7 58.5 (40–92)	59.6 58.0 (40–98)
Household income, 1,000 DKK*: median**	300–399	300–399	200–299	300–399	300–399	300–399	300–399
Proportion female	49.6%	56.5%	56.9%	50.4%	55.1%	55.0%	53.9%
Prevalence of hypercholesterolaemia	16.8%	22.3%	19.1%	19.2%	17.0%	16.6%	18.6%
Prevalence of previous heart attack	5.6%	7.8%	3.4%	7.1%	5.6%	7.1%	6.0%

*£1.00 = DKK11, **(minimum = (<DKK100,000, maximum- = DKK799,000+)

On average 58% of respondents accepted the hypothetical drug treatment, 27% rejected it and 15% were uncertain (Table 2). There was a significant trend towards increasing acceptance to the treatment with increasing RRR for respondents presented with baseline risk (χ^2^-test for trend, *p *= 0.02), but the trend was not sustained when RRR = 10 was excluded from the analysis (χ^2^-test for trend, *p *= 0.11). No significant trend was found for respondents who were not presented with baseline risk, even when RRR was included. (*p *= 0.45) (Table 2). Presence or absence of baseline risk did not influence the respondents' general willingness to accept the treatment (Table 2).

**Table 2:** Preferences for heart attack prevention according to its effectiveness in terms of RRR

**"Would you choose to take such a drug?"**							
**Baseline risk presented**				**Baseline risk not presented**			
	
**RRR**	**Yes (%)**	**No (%)**	**Uncertain (%)**	**RRR**	**Yes (%)**	**No (%)**	**Uncertain (%)**
	
10 (n = 128)	50.0	37.5	12.5	10 (n = 126)	47.6	34.9	17.5
20 (n = 152)	55.9	28.3	15.8	20 (n = 110)	65.5	20.0	14.5
30 (n = 130)	53.8	33.1	13.1	30 (n = 139)	61.9	20.1	18.0
40 (n = 139)	58.3	25.9	15.8	40 (n = 117)	57.3	26.5	16.2
50 (n = 130)	60.0	25.4	14.6	50 (n = 106)	60.4	29.2	10.4
60 (n = 127)	63.8	22.8	13.4	60 (n = 115)	56.5	20.9	22.6
	
Total (n = 806)	56.9	28.8	14.3	Total (n = 713)	58.1	25.2	16.7

Among the respondents, 76% reported that it was not difficult to understand RRR, while 17% found it somewhat difficult, 4.5% very difficult and 2.4% found it impossible to understand. There was no difference in reported understanding of RRR across respondents presented or not presented with baseline risk information (24.2% *versus *24.0%, *p *= 0.92). Respondents, who reported no difficulties understanding the concept, were more likely to accept the hypothetical treatment irrespective of RRR-level and whether baseline risk had been presented.

Low income was associated with high prevalence of self-reported hypercholesterolaemia, high self-reported previous heart attack, and difficulties with understanding the RRR concept (all χ^2 ^trend analyses *p *< 0.001).

According to a logistic regression analysis (Table 3), respondents presented with a RRR of 10 were less likely to accept the treatment when using RRR = 60 as reference, while there was no difference for the other groups. Those diagnosed with hypercholesterolaemia or heart disease were more likely to accept the hypothetical treatment, as were respondents who did not find it difficult to understand RRR. Further, the odds for accepting the hypothetical treatment increased with higher household incomes, but decreased with higher levels of education.

**Table 3:** Multiple logistic regression analysis of the odds for accepting the hypothetical treatment (0 = no or uncertain, 1 = yes)

**Variable**	**Odds ratio**	**(95% CI)**
Baseline risk presented (0 = no, 1 = yes)	1.14	0.90–1.44

RRR (reference: RRR = 60)		
RRR = 10	0.60*	0.39–0.90
RRR = 20	1.03	0.69–1.55
RRR = 30	0.91	0.60–1.38
RRR = 40	0.97	0.64–1.46
RRR = 50	1.05	0.69–1.59
Level of education (1–8; 1 = lowest, 8 = highest)	0.93*	0.87–0.99
Annual household income (1–8; 1 = <DKK 100,000, 8 = DKK 799,000+)	1.09*	1.01–1.17
Suffer from hypercholesterolaemia (0 = no, 1 = yes)	1.78*	1.27–2.49
Previously experienced heart attack (0 = no, 1 = yes)	2.38*	1.27–4.47
Respondents finding RRR difficult to understand (0 = no, 1 = yes)	0.33*	0.25–0.44

N = 1245, Respondents excluded from the analysis due to missing data; 274 (15.4%). -2log likelihood = 1585.577, χ^2 ^= 102.344, *p *< 0.001.

*: *p *< 0.05

With the purpose of possibly confirming higher sensitivity to RRR when baseline risk was presented in numeric terms (as indicated by the trend analysis, Table 2), logistic regression was performed including an additional interaction variable: RRR*baseline risk. This interaction variable was not significant in the logistic regression, and an effect of baseline risk on effect of RRR information could not be confirmed.

## Discussion

It is conceivable that effectiveness, along with costs and side effects, is a core issue when people consider a potential therapy. We therefore expected the consent to the hypothetical therapy to increase with increasing effectiveness. The results of the study, however, indicate only weak and inconsistent effects. In theory, information on baseline risk is necessary to interpret RRR correctly, but such information had only little impact on the choices respondents made in this study. Hence, in a hypothetical situation, respondents are only slightly influenced by a difference between 10% versus 60% RRR of the given drug treatment, and RRR-effect was not influenced by presentation of baseline risk.

Presentation of baseline risk did not affect respondents' general acceptance of the hypothetical treatment according to the logistic regression analysis (Table 3). This was in contrast to our expectations that fewer respondents would accept the treatment when presented with baseline risk, because presentation of baseline risk makes the small absolute benefit more evident. These results do however accord with previous evidence of baseline neglect [10,11]. A specific problem associated with our design is also that one could argue that base-line risk was – in some form – presented to all respondents, and it is conceivable that 1% baseline risk is perceived as about the same risk as "slightly increased risk of heart attack". I.e. an explanation could be that baseline risk was fairly low. Another explanation may simply be lack of numeracy skills among some respondents.

Gyrd-Hansen et al [8] explored whether presentation of baseline risk had an effect on treatment choices and found that lower baseline risk and thus higher RRRs (holding ARR constant) resulted in greater acceptance rates. The current study could not extend this previous finding to a more general effect of the presence of baseline risk information. This may be due to a different study design, where we test for the effect of presence/absence of baseline risk information rather than varying baseline risk. Alternatively, the reason may be that the cognitive burden of calculating ARR on the basis of RRR and baseline risk is greater than that of calculating RRR on the basis of ARR and baseline risk. The ideal design would involve many levels of both RRR and baseline risk. This would inflate the necessary number of respondents, a design that was not feasible in the present study.

We found a positive relationship between self-reported understanding of the RRR concept and acceptance of the treatment, which suggests that respondents, who think they understand the concept, are more likely to opt for the treatment. There was no association, however, between self-reported understanding of RRR and presentation of baseline risk. This suggests that respondents' perceived understanding does not depend on whether they have received sufficient information to make a fully informed decision. This result is important when communicating the effect of a medical treatment, since those who say that they understand the concept off RRR do not necessarily understand that they need baseline risk to use RRR properly.

In several studies [6,13-18], respondents have been more likely to accept an intervention when its effectiveness was presented in terms of RRR rather than NNT. In the aforementioned study by Kristiansen et al [7], the acceptance rate was on average 80% for NNT while it was only 60% in the present study of RRR with a similar design. In both studies, the price of the drug, the side effects and the need for medical follow-up were quite similar. When our study and the study by Kristiansen [7] seemingly contradict previous findings [6,13-18], the explanation may be found in small differences in the format of the two studies. Lay people may be quite sensitive to small differences in presentation of disease, side effects, and price while they may be insensitive to considerable differences in treatment effectiveness.

In another study with similar design [19], respondents' preferences for a therapy were strongly influenced by treatment effectiveness expressed as postponement of hip fracture. The question is then why preferences for therapy were little influenced in this study off RRR. The explanation is probably that laypeople, who are not familiar with relative risks, are almost unable to get meaningful information out of the numbers even if they understand the numbers themselves. In the absence of real understanding of effectiveness, people will tend to use other information, which they do understand. Such pieces of information may be type of disease (for example osteoporosis versus hypercholesterolaemia), type of outcome (heart attack versus death), costs etc as found in a study concerning NNT [6].

Finally, the significant effect of income on acceptance of the treatment suggests that respondents may have considered and applied the cost information when responding. The respondents appear to have understood and considered some of the information presented to them, while remaining insensitive to the risk information. However, it is also possible that other factors may be related to income, e.g. education or health literacy, which may affect respondents to trust the medical system or be more willing to take medications. This observation emphasises the problems associated with communicating this type of information to lay people.

### Limitations

Some of the findings were unexpected, but they should, however, be seen against the limitations of the study. It is likely that a discrepancy exists between a choice made in a hypothetical situation and a choice made in a real life situation. A similar problem exists when comparing the agreement of doctors' responses to written simulations compared to their responses to actual clinical encounters [20], and the validity of hypothetical scenarios is not established. More research is needed to decide whether theoretical situations can measure actual behaviour. Ideally, the study should have been undertaken among real patients, but earlier studies on NNT would not indicate that patients are better able to handle risk information than healthy lay persons [21].

The way we presented the RRR percentages to the respondents is somewhat ambiguous in that the numbers (10%–60%) may be interpreted as absolute risk reductions. We believe that this potential misunderstanding was not a major problem in the study. First, 10% or more ARR is reduction is unrealistic for most interventions although respondents may not know this. Second, the responses were about the same whether or not respondents were presented with baseline risk. Those who were informed about baseline risk would know that the numeric information was relative, not absolute.

Further aspects of whether the respondents understood the RRR concept could have been explored by e.g. making a direct check asking the respondent to recall the exact RRR percentage, and asked the respondents of their perceived effectiveness of the drug.

## Conclusion

In conclusion, respondents were in general insensitive to RRR information whether baseline risk information was presented or not. This finding is in line with previous studies, which suggest that lay people have difficulties in understanding risk information, but stands in contrast to studies that show that lay people can differentiate between level of effectiveness when it is presented in terms of postponement of adverse events such as death or hip fracture [21]. Informed and shared decision making requires methods of communicating intervention effectiveness that is well understood and applied by patients in the decision making process. Information on risk reduction in absolute terms is necessary to make rational choices, but relative risks may be frequently used in practice. This study represents an additional argument against the use of RRR when informing patients about treatment effectiveness.

## Conflict of interests

The authors declare that they have no competing interests.

## Authors' contributions

DG–H, ISK, JN and JBN designed the study, and LS performed the analyses and drafted the manuscript. All authors read and approved the final manuscript.

## Pre-publication history

The pre-publication history for this paper can be accessed here:


